# Relationship of Oxidative Stress as a Link between Diabetes Mellitus and Major Depressive Disorder

**DOI:** 10.1155/2019/8637970

**Published:** 2019-03-03

**Authors:** Gislaine Z. Réus, Anelise S. Carlessi, Ritele H. Silva, Luciane B. Ceretta, João Quevedo

**Affiliations:** ^1^Translational Psychiatry Laboratory, Graduate Program in Health Sciences, Health Sciences Unit, University of Southern Santa Catarina, Criciúma, SC, Brazil; ^2^Programa de Pós-graduação em Saúde Coletiva, Universidade do Extremo Sul Catarinense, Criciúma, SC, Brazil; ^3^Center of Excellence on Mood Disorders, Department of Psychiatry and Behavioral Sciences, McGovern Medical School, The University of Texas Health Science Center at Houston (UTHealth), Houston, TX, USA; ^4^Translational Psychiatry Program, Department of Psychiatry and Behavioral Sciences, McGovern Medical School, The University of Texas Health Science Center at Houston (UTHealth), Houston, TX, USA; ^5^Neuroscience Graduate Program, Graduate School of Biomedical Sciences, The University of Texas Health Science Center at Houston (UTHealth), Houston, TX, USA

## Abstract

Both conditions, major depressive disorder (MDD) and diabetes mellitus (DM) are chronic and disabling diseases that affect a very significant percentage of the world's population. Studies have been shown that patients with DM are more susceptible to develop depression, when compared to the general population. The opposite also happens; MDD could be a risk factor for DM development. Some mechanisms have been proposed to explain the pathophysiological mechanisms involved with these conditions, such as excess of glucocorticoids, hyperglycemia, insulin resistance, and inflammation. These processes can lead to an increase in damage to biomolecules and a decrease in antioxidant defense capacity, leading to oxidative stress.

## 1. Introduction

### 1.1. Diabetes Mellitus

Diabetes mellitus (DM) is characterized by hyperglycemia due to changes in the production or action of insulin; the chronicity of this condition is associated with damage, dysfunction, and insufficiency of target systems such as cardiovascular and central nervous systems [1]. The physiopathology of DM is related to changes in *β*-pancreatic cells that compromise the synthesis and secretion of insulin, together with resistance to the action of insulin in peripheral tissues. Insulin secretion is controlled by several factors, including nutrients, hormones, and neural factors [2]. One of the roles of insulin is to influence inflammatory reactions by it acting on oxidative stress and in the release of cytokines [3]. The inflammatory component in the physiopathology of DM is evidenced by the involvement of the factor nuclear kappa B (NF-*κ*B), which is one of the transcription factors that control the production of proinflammatory cytokines. The NF-*κ*B pathway binds the inflammatory and metabolic responses and represents a point of connection for a better understanding of metabolic diseases [4]. In addition, chronic conditions of low-grade inflammation appear to play an important role in the pathogenesis of renal failure, one of the consequences of DM [5]. Hyperglycemia, a frequent condition in DM, is related to cellular and tissue damage, due to changes in cell signaling, gene transcription, and protein and lipid changes [6].

### 1.2. Major Depressive Disorder

Major depressive disorder (MDD) has high morbidity, and nearly 350 million people are affected worldwide. The physiopathological mechanism is not widely understood, but is believed to have a multifactorial origin, involving dysfunction in multiple brain areas such as the hippocampus, prefrontal cortex, nucleus accumbens, and amygdala [7]. Moreover, MDD pathophysiology is associated to an inflammatory process due to microglial activation, elevated cytokine release, and increased oxidative stress, with astrocyte atrophy and alteration in glutamatergic system regulation, which may lead to local damage [8]. These processes may also activate the enzyme indoleamine 2,3-dioxygenase, diverting tryptophan to the kynurenine pathway, causing the production of active neurotoxic metabolites [9]. In fact, it is known that microglial cells regulate the activation and progression of several neuroimmune pathways that are mediated by macrophages, growth factors, cytokines, and others. In addition, they also initiate the formation of intracellular multiprotein complexes, the inflammasomes, which in turn cleave precursor forms of interleukin-1*β* (IL-1*β*) in its active form [10]. The inflammatory process, when exacerbated, can cause a significant increase in the production and expression of proinflammatory cytokines such as tumor necrosis factor-*α* (TNF-*α*) and IL-1*β*, as well as reactive species of oxygen (ROS) and nitric oxide, contributing to the neuroinflammatory and neurodegenerative processes associated with psychiatric disorders, including MDD [11]. In another recent study, it was identified that MDD, associated or not to posttraumatic stress disorder, presents changes in the cytokines and increased oxidative stress [12], thus demonstrating that the association of several factors contributes to the pathophysiology of MDD.

### 1.3. Oxidative Stress

The term oxidative stress is used to characterize the imbalance between the production of ROS and antioxidant defenses. Elevated levels of ROS cause damage to lipids, proteins, and DNA [13], and it is associated with several diseases including cancer, DM, cardiovascular, neurodegenerative diseases, and MDD [14].

Under normal physiological conditions, there is formation of ROS and reactive nitrogen species (RNS) that act as a messengers and also regulate intracellular signal transduction pathways involved with survival and cell death, being removed by several mechanisms of antioxidant defense, such as catalase and superoxide dismutase enzymes [15]. However, when in excess, they are harmful to the metabolism, mainly because of being able to inactivate important cellular molecules which are necessary for the regulation and homeostasis [16]. Antioxidants are a defense system for the body against these and all free radicals. They act by eliminating or by preventing their transformation into products that are less toxic to cells [17].

There is an important relationship between some diseases and oxidative stress, because they participate in vital processes, such as inflammation, glucose homeostasis, and cell survival [18]. In DM, hyperglycemia induces increased oxidative stress through several biochemical processes [19], including the glucose self-activation, increase of glycation and diacylglycerol, and the activation of protein kinase C and polyol pathways. Also, this process causes the progression and complications of DM due to increased free radicals and decreased antioxidant enzymes leading to an increase in lipid peroxidation [20]. A significant increase in oxidative stress in diabetic patients compared to controls was observed, but this change appears to be more evident during disease progression and complications [21, 22].

Oxidative stress also may be related to some psychiatric disorders. There is evidence that in the MDD patients, excess of ROS may be a relevant mechanism related to immune activation [23], increased oxidation of monoaminergic neurotransmitters [24], and lipid peroxidation [25]. Furthermore, in MDD there is also a decrease in important antioxidant substances as well as a lower activity of the antioxidant enzymes [26].

### 1.4. Diabetes Mellitus, Major Depressive Disorder, and Oxidative Stress

Studies have shown that patients with DM have a higher risk to develop MDD, when compared to the general population [27–30]. Patients with MDD, as well as the use of antidepressant drugs, could be risk factors for the development of DM [31–34]. In addition, depression in patients with DM is a major cause of poor self-care, which are very important for these patients to avoid future complications, for example, renal, ocular, and neurological damage [35].

It is believed that the glucose accumulation in the extracellular space due to DM can cross the blood brain barrier (BBB) and affect specific brain areas involved with memory and mood regulation [36, 37]. On the other hand, MDD may be correlated with insulin resistance due to higher levels of glucocorticoid and a decrease in insulin sensitivity [38, 39]. It was proposed by Watson [40] that DM and other diseases such as cancer and dementias are accelerated or caused by failure of the endoplasmic reticulum to generate sufficient oxidative redox potential for disulphide bonds to be formed. Indeed, genomics, epigenomics, and exposomics methods are suggested to characterize redox components and their functional organization in health and disease [41].

The pathophysiological mechanism involved when both DM and MDD are together is still not clear. One of these mechanisms could be related to the oxidative stress (Table 1). In fact, oxidative stress plays an important role in the development and progression of DM due to higher free radical production, damage to cell constituents, and impairment in the antioxidant defense enzymes, such as superoxide dismutase and catalase [42, 43]. MDD also is characterized by activated oxygen and nitrogen species pathways, leading to lipid, protein, and DNA damage [44–47].

Experimental studies have been shown that alloxan-diabetic rats displayed a depressive-like behavior in the forced swimming test [48, 49], while the treatment with the antidepressant imipramine [48] and with the antioxidant N-acetylcysteine (NAC) [49] was able to reverse the depressive-like behavior, thus showing that both antidepressant and antioxidant could improve depressive behavior induced by the animal model of diabetes. The treatment with clonazepam, a positive GABA_A_ receptor modulator, alone or in combination with insulin also reversed the depressive-like behavior in diabetic rats [50–52]. Interestingly, the treatment with insulin and clonazepam was able to restore the antioxidant status in the brain of diabetic rats [52]. A study carried out by Tang et al. [53] demonstrated that hydrogen sulfide (H2S), a signaling molecule in the brain, with antioxidant activity was able to reverse the depressive-like behavior in streptozotocin- (STZ-) induced diabetic rats. The authors suggested that this behavioral change was associated to a reduction in oxidative stress in the hippocampus. Recently, Shivavedi et al. [54] showed that a combination treatment with metformin and ascorbic acid reduced the depressive-like behavior, oxidative stress and inflammation, and elevated monoamine levels in STZ-induced diabetic rats. It was suggested that the antidepressant effects exercised by metformin and ascorbic acid in diabetic rats were associated with a reduction in blood glucose and oxidative stress and increased plasma insulin levels [54]. Ascorbic acid, a natural antioxidant, was proposed as a potential strategy against comorbid depression-like behavior in diabetic rats. It was revealed that ascorbic acid treatment reduced the depressive behavior in STZ-nicotinamide-induced diabetic rats [55]. Also, it was demonstrated that ascorbic acid reduced oxidative stress, hyperglycemia, and inflammation [55, 56]; on the other hand, positive results with *Aloe vera* treatment were found, which has antioxidative, neuroprotective, and antidiabetic effects. The study revealed that *Aloe vera* displayed antidepressant effects in STZ-induced diabetic rats, and these effects were suggested to be related to hypoglycemic and antioxidant properties of *Aloe vera* in the hippocampus [56].

Some studies have reported a potential therapeutic for ebselen, a glutathione peroxidase mimetic and which can contribute to regulation of cell function [57, 58]. Experimental studies revealed that treatment with ebselen reduced diabetes-associated atherosclerosis in apolipoprotein E/GPx1 double-knockout mouse [59], prevented islet apoptosis, and preserved the *β*-cell mass and function in Zucker diabetic fatty (ZDF) rats [60]. Also, ebselen treatment in human erythrocytes from patients with uncontrolled diabetes exerted glycation-inhibiting properties [61]. Contrarily, a randomized, crossover trial with DM patients did not show improvement in the oxidative stress profile and it did not affect the endothelium-dependent vasodilation [62]. There are no studies evaluating the effects of ebselen in depression; however, a study demonstrated that ebselen due to its capacity to inhibit the inositol monophosphatase could be an alternative treatment for bipolar disorder, comparable to lithium [58]. Future studies evaluating the efficacy of ebselen in depression and DM comorbidity could be interesting.

A human study with MDD and bipolar disorder patients revealed no association with mood disorder symptoms and insulin resistance or increased glucose toxicity [63]. However, the same study demonstrated effects for severity of mood disorders on glucose levels and in the number of mood episodes on glucose toxicity. In addition, *β*-cell function and insulin resistance were associated with immune-inflammatory, ROS, and RNS pathways, which in turn induced glucose toxicity [63]. Contrarily, a recent cohort study revealed that higher levels of systemic oxidative stress, marked by DNA/RNA damage from oxidation (8-oxodG/8-oxoGuo) in patients with DM, were not associated with higher risk for psychiatric diseases, such as unipolar depression, anxiety, bipolar disorder, and schizophrenia [64]. Discrepancies in these studies may be related to the type of marker studied, study time, and psychiatric disorder conditions analyzed.

## 2. Conclusion

The imbalance between ROS formation and the antioxidant system can result in several pathological alterations that are related to both psychiatric and metabolic diseases, and these changes are evident mainly in progressive and chronic pathologies such as DM and MDD.

Although few studies have evaluated the relationship of oxidative stress when MDD and DM are present in the same patient, oxidative stress oxidative redox potential may be the key factor in triggering comorbidities such as MDD associated with DM and vice versa; nevertheless, many other factors such as inflammation, hyperglycemia, and insulin resistance are also involved, although all these conditions increase the levels of oxidative stress.

Further studies evaluating medications with antidepressant and antioxidant effects that can reduce oxidative stress may be clinically important to prevent comorbid MDD in DM condition.

## Figures and Tables

**Table 1:** Summary of changes associated to oxidative stress in diabetes mellitus and major depressive disorder.

Species/model	Damage	Antioxidant effect	Reference
Alloxan-diabetic rats	Depressive behavior	*N-*Acetylcysteine and imipramine displayed antidepressant effects	[48, 49]
Diabetic rats	Depressive behavior and oxidative stress	Clonazepam and insulin reversed the depressive behavior and restored the antioxidant status	[50, 52]
STZ-diabetic rats	Depressive behavior	Hydrogen sulfide induced antidepressant effects	[53]
STZ-diabetic rats	Depressive behavior, oxidative stress, and inflammation	Metformin plus ascorbic acid reduced the depressive behavior and had antioxidant and anti-inflammatory effects	[54]
STZ-nicotinamide-diabetic rats	Depressive behavior	Ascorbic acid had antidepressant effects, reduced oxidative stress, and inflammation	[55, 56]
STZ-diabetic rats	Depressive behavior	*Aloe vera* displayed antidepressant, antioxidant, and antidiabetic effects	[56]
MDD and bipolar disorder patients	Severity of symptoms was associated to glucose levels and the number of episodes to glucose toxicity	—	[63]
